# Dr. Moseley's Account of a Recent Case of Hydrophobia

**Published:** 1807-12

**Authors:** Benjamin Moseley

**Affiliations:** Chelsea Hospital


### Dr. Moseley's *Account of a recent* Case *of* Hydrophobia.

This afternoon, at three o'clock, Mrs. Metcalfe, No. 23, Compton Street, brought her son, Mr. Frederick Michael Metcalfe, to me for advice, at my house in Albany, Piccadilly.

He informed me, that he was attacked about four o'clock yesterday morning with a difficulty in swallowing any liquid, which he first perceived when he attempted to drink some porter, the remains of half a pint, which he had on the preceding evening. He said, wheu he put the pot to his mouth, something rose in his throat, and choaked him. He swallowed, as he thought, about a tea spoonful, and then was seized with a trembling, and cramp in his arms and legs, and a sensation of pricking, as if pins, or needles, were run into his flesh. His appetite failed him on Saturday last. Yesterday he ate a small piece of mutton, which made him sick at his stomach. He has eaten nothing this day; though he said he could swallow any thing, except it were in a liquid form; but has no desire for food. He said he was attacked on Thursday last with a violent pain in his right arm, from his shoulder to the ends of his fingers. This pain left him on Saturday night. He rubbed the arm with hartshorn and oil, and wrapped it up with flannel, on Saturday.

Mrs. Metcalfe informed me, that on his seeing any liquid poured out for him to drink, even before he takes hold of the pot, or cup, he begins to tremble, and the choaking choaking seizes him. She said, in attempting to drink, he becomes convulsed, his eyes look glassy, and he stares in an unusual and frightful manner.

The Case thus clearly demonstrated, I desired Mrs. Metcalfe to go with me into another room. I did this that I might not alarm her son, by questions necessary for further information. Neither Mrs. Metcalfe nor her son had the slightest suspicion of the cause, or the nature, of this dreadful calamity.

I asked Mrs. Metcalfe whether her son had been lately bitten by any dog? The very question so much alarmed her, that she was for a few minutes in a state of distraction. When she was able to speak, she exclaimed, with a loud shriek, that he had been bitten in the hand by a dog in the summer. As soon as she became calm, and composed, we returned to her son.

On interrogating him, he informed me, that in the beginning of July last, there were two dogs fighting desperately in the street opposite his mother's house; and he observing one of them had one of his eyes torn out, and the other dog likely to kill him, endeavoured to part them; but on taking hold of the dog he wished to rescue from the fury of the other, he received a bite from him on his right hand. Two of the dog's teeth penetrated the outside of the hand, but the palm of the hand was considerably wounded. This wound was dressed with Friar's balsam and poulticed, and was cured in a week or ten days.

I examined his hand.—There was a small degree of redness remaining, but no heat, or pain, where the wound had been in the palm of his hand, and no vestige whatever, on the outside where the teeth had been. There was nothing observable in his throat, differing from its natural state; nor any increase of saliva. Pulse 88, rather feeble, and not quite regular. He had no thirst. He told me his choaking seemed to him as arising from wind; and that he always discharged a great deal from his throat whenever he attempted to swallow. He said he took some dillseed water last night, and thought it relieved him; but never could get down more than a tea-spoon full at a time, and that with great difficulty. In one attempt to swallow some of this water, he was so choaked and convulsed, that he would have fallen into the fire, his mother told me, if she had not saved him. I gave him some water in a pint pot twice; each time he swallowed about a tea-spoon full, and both times was choaked, and convulsed, with a wild staring in his eyes, and a trembling all over him; and immediately after the effort of swallowing, he made a hideous noise. The second time I gave him the water, I was much alarmed; I thought it would have occasioned a fatal convulsion. It is impossible to describe a sound; and I can compare the noise he made, which was from repeated spasmodic contractions of the organs of respiration, to nothing but to that sort of stifled barking which dogs sometimes make, when disturbed in their sleep; or to the hoarse, short barking of a drover's dog. When he took the pot in his hand, he fell into a tremor, held down his head, and was in great distress; he kept the pot in his hand a few seconds before he could summon courage to lift it to his mouth; after which I took it from him, as from his agony he could not hold it. He bore the sight of the water in the pot, while it was in my hand, when it was not offered him to drink; but when I brought a large bason filled with water, and put it before his eyes, he seemed frightened; and when I agitated the water near him, he was instantly attacked with what he called "*the wind rising in his throat*," trembling, and that hoarse, faucial noise before mentioned. He entreated me not to order any medicine for him in a liquid form, as he said he could not take it; and the attempt, he was certain, would kill him. He said he could swallow any solid substance. I put this to the proof; and, as he had been costive for several days, I gave him four aperient pills, which he swallowed one at a time, but with some difficulty.—He had now been with me three quarters of an hour, when he and Mrs. Metcalfe left Albany, with the best advice I could give, and walked back to Compton-street. From his appearance, and conversation, no person would have thought there was any indisposition about him. His voice and speech had suffered no alteration. He was in the eighteenth year of his age; a very fine youth in mind, as well as in person. His humanity here was his misfortune. With what grief did I see him depart from Albany, with his poor mother, knowing, as I did, that he had but a few hours to live! I visited him at eight o'clock in the evening. Pulse 110, and very feeble. I gave him some water. In attempting to drink, the usual consequences,—choaking, wildness in the eyes, and the noise in the throat, followed. The pills operated about nine o'clock, several times. About ten o'clock he became so violently convulsed, that four young men, his brothers, could scarcely keep him in his bed; but he made no attempt to bite any person. He began also to foam at the mouth, with white froth.

froth. The quantity of this froth was so great, as to require many towels and handkerchiefs, in wiping it from his mouth. At this period he likewise became delirious at intervals, but at times in his perfect senses; and complained, though in a very warm room, of being cold, and begged to be kept warm. In this condition he continued until one o'clock on the following morning, when, from his violent convulsive exertions and struggling, he was entirely exhausted, and remained calm and quiet afterwards.

He expired at a quarter before two, 18 weeks from the time of the accident; 46 hours from the commencement of the *hydrophobia*; and ten hours after I first saw him.

BENJAMIN MOSELEY.

*Chelsea Hospital, November* 9, 1807.

